# Supplementary material for: Pleiotropic Effects of APOB Variants on Lipid Profiles, Metabolic Syndrome, and the Risk of Diabetes Mellitus
Source: Int J Mol Sci. 2022 Nov 29;23(23):14963. doi: 10.3390/ijms232314963 (PMC9735756; doi:10.3390/ijms232314963)
Supplement: Supplementary file 1 [file ijms-23-14963-s001.zip › APOB SNPs Supplementary 20221005.pdf]

Supplementary Method

Definitions of metabolic syndrome and diabetes mellitus

Diabetes mellitus (DM) was defined as a fasting plasma glucose level of ≥126 mg/dL, a glycohemoglobin value of ≥6.5%, or a self-reported history of DM. Because medication histories were unavailable, metabolic syndrome characteristics were based on the recent update of the third report of the National Cholesterol Education Program's Adult Treatment Panel III criteria [1] with modifications. Participants with three or more of the following attributes are typically defined as having metabolic syndrome: (1) BP of ≥130/85 mmHg or a history of hypertension; (2) triglyceride level of ≥150 mg/dL; (3) high-density lipoprotein cholesterol level of <40 mg/dL for men or <50 mg/dL for women; (4) fasting plasma glucose of ≥100 mg/dL or a history of DM; and (5) waist circumference of >90 cm for men or>80 cm for women.

Reference:

1. Grundy SM, Cleeman JI, Daniels SR, Donato KA, Eckel RH, Franklin BA, et al. Diagnosis and management of the metabolic syndrome: an American Heart Association/National Heart, Lung, and Blood Institute Scientific Statement. Circulation. 2005; 11: 2735–52.

Supplementary Table S1. *APOB* exonic mutations in Taiwan Biobank participants: Data derived from the whole genome sequence.

Chr	SNPs	Position (GRCh37)	Ref/Alt	HWE	MAF	Exon number	Exonic Function	mRNA sequence	AA Change.	LDL cholesterol		
										Beta	se	*p* value
2	rs144467873	21229161	G/A	0.2916	0.0019	26	Non-synonymous mutation	c.C10579T	p.Arg3527Trp	0.1862	0.047	7.75×10^{-5}
2	rs676210	21231524	A/G	0.6796	0.2732	26	Non-synonymous mutation	c.C82169T	p.Pro2739Leu	-0.0062	0.005	0.2105
2	rs679899	21250914	A/G	0.2891	0.1482	14	Non-synonymous mutation	c.C1853T	p.Ala618Val	-0.0009	0.006	0.8827
2	rs13306194	21252534	G/A	0.0494	0.1425	12	Non-synonymous mutation	c.C1594T	p.Arg532Trp	-0.0112	0.0065	0.0839
2	rs1367117	21263900	G/A	0.8006	0.1320	4	Non-synonymous mutation	c.C293T	p.Thr98Ile	0.0091	0.0064	0.1544

Chr: chromosome, SNP: single nucleotide polymorphism, Ref/Alt: reference/alternate allele, HWE: HWE: Hardy-Weinberg equilibrium, MAF: minor allele frequency, mRNA: messenger RNA, AA: amino acid, se: standard error.

Participants were analyzed after the exclusion of those with a history of hyperlipidemia.

p value: adjusted for sex, age, BMI and current smoking.

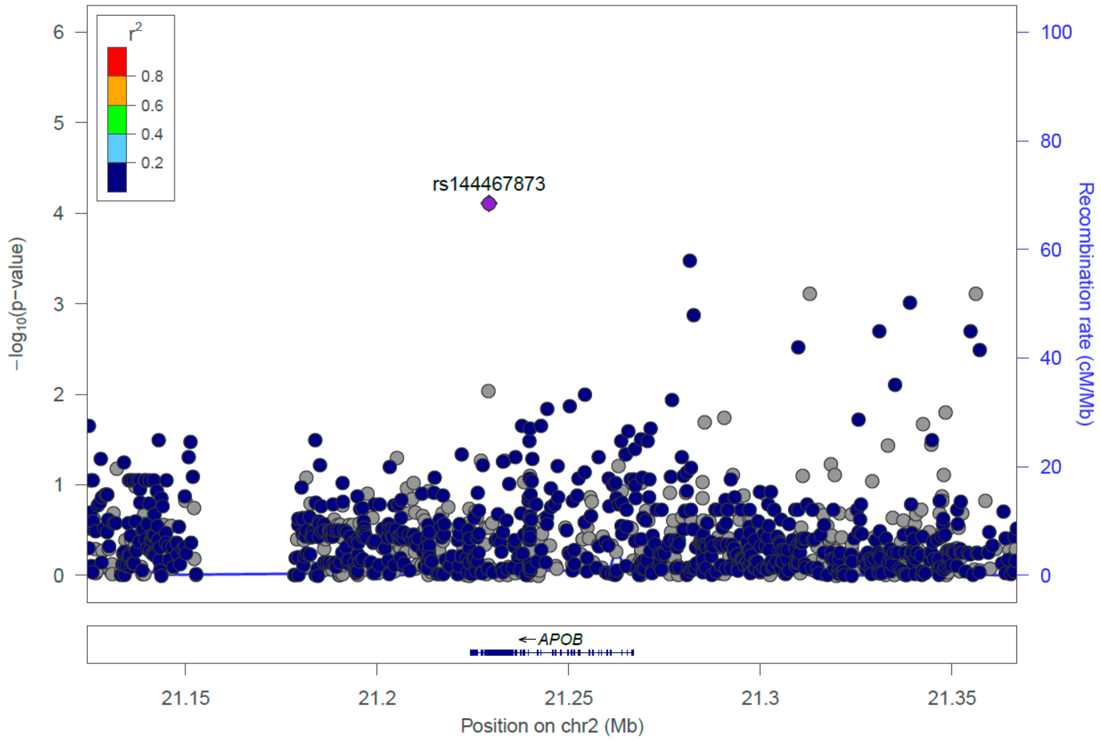

Supplementary Figure S1. Regional association analysis between *APOB* variants and LDL cholesterol levels in Taiwan Biobank participants with whole genomic sequence data.

Supplementary Table S2. Association between *APOB* locus non-coding variants and serum lipid profile and metabolic syndrome.

Genetic variants	Genotypes			Beta	se	*p* value
APOB rs35131127	TT (88,660)	TC (16,879)	CC (777)			
Total cholesterol (mg/dL)	193.0 (171.0 - 217.0)	194.0 (172.0 - 218.0)	197.0 (175.0 - 219.0)	0.0029	0.0006	7.31×10^{-7}
LDL cholesterol (mg/dL)	119.0 (99.0 - 140.0)	120.0 (101.0 - 142.0)	124.0 (103.0 - 144.0)	0.0048	0.0009	4.82×10^{-8}
Non-HDL cholesterol (mg/dL)	137.0 (116.0 - 161.0)	139.0 (118.0 - 164.0)	143.0 (122.0 - 165.0)	0.0068	0.0008	1.00×10^{-17}
HDL cholesterol (mg/dL)	54.0 (45.0 - 63.0)	53.0 (45.0 - 62.0)	52.0 (43.0 - 61.0)	-0.0072	0.0007	1.05×10^{-24}
Triglyceride (mg/dL)	90.0 (63.0 - 132.0)	93.0 (66.0 - 138.0)	99.0 (70.0 - 144.0)	0.0190	0.0016	7.31×10^{-31}
Remnant cholesterol (mg/dL)	16.0 (11.0 - 23.0)	17.0 (12.0 - 24.0)	18.0 (12.0 - 25.0)	0.0153	0.0020	2.43×10^{-14}
Metabolic syndrome (%)	25.32% (24,238)	26.49% (4852)	28.71% (242)	0.1051	0.0194	5.94×10^{-8}
APOB rs56213756	CC (82,301)	CG (22,644)	GG (1504)			
Total cholesterol (mg/dL)	193.0 (171.0 - 217.0)	194.0 (172.0 - 217.0)	196.0 (174.0 - 218.0)	0.0020	0.0005	0.0001
LDL cholesterol (mg/dL)	119.0 (99.0 - 141.0)	120.0 (100.0 - 141.0)	122.0 (102.0 - 143.0)	0.0030	0.0008	0.0001
Non-HDL cholesterol (mg/dL)	137.0 (116.0 - 161.0)	139.0 (117.0 - 163.0)	142.0 (121.0 - 165.0)	0.0052	0.0007	2.54×10^{-14}
HDL cholesterol (mg/dL)	54.0 (45.0 - 63.0)	53.0 (45.0 - 63.0)	53.0 (44.0 - 62.0)	-0.0062	0.0006	2.23×10^{-24}
Triglyceride (mg/dL)	90.0 (63.0 - 132.0)	93.0 (66.0 - 137.0)	96.0 (69.0 - 142.0)	0.0161	0.0014	1.04×10^{-29}
Remnant cholesterol (mg/dL)	16.0 (11.0 - 23.0)	17.0 (12.0 - 24.0)	17.0 (12.0 - 25.0)	0.0167	0.0017	8.14×10^{-22}
Metabolic syndrome (%)	25.24% (22,436)	26.44% (6482)	28.01% (458)	0.0966	0.0168	8.96×10^{-9}
APOB rs1318006	AA (81,905)	AG (22,333)	GG (1470)			
Total cholesterol (mg/dL)	193.0 (171.0 - 217.0)	193.0 (171.0 - 217.0)	196.0 (174.8 - 219.0)	0.0019	0.0005	0.0001
LDL cholesterol (mg/dL)	119.0 (99.0 - 140.0)	120.0 (100.0 - 141.0)	122.5 (102.8 - 143.0)	0.0030	0.0008	0.0001
Non-HDL cholesterol (mg/dL)	137.0 (116.0 - 161.0)	139.0 (117.0 - 163.0)	142.0 (121.0 - 165.0)	0.0052	0.0007	6.32×10^{-14}
HDL cholesterol (mg/dL)	54.0 (45.0 - 63.0)	53.0 (45.0 - 62.0)	53.0 (44.0 - 62.0)	-0.0064	0.0006	2.33×10^{-25}
Triglyceride (mg/dL)	90.0 (63.0 - 132.0)	93.0 (66.0 - 137.0)	96.0 (69.0 - 141.0)	0.0161	0.0014	2.98×10^{-29}
Remnant cholesterol (mg/dL)	16.0 (11.0 - 23.0)	17.0 (12.0 - 24.0)	17.0 (12.0 - 25.0)	0.0164	0.0017	5.21×10^{-21}
Metabolic syndrome (%)	25.24% (22,332)	26.45% (6396)	27.44% (438)	0.0940	0.0169	2.76×10^{-8}
APOB rs4665709	GG (91,312)	GA (14,119)	AA (509)			
Total cholesterol (mg/dL)	193.0 (171.0 - 217.0)	193.0 (172.0 - 217.0)	196.0 (173.0 - 216.5)	0.0016	0.0006	0.0133
LDL cholesterol (mg/dL)	119.0 (99.0 - 140.0)	120.0 (100.0 - 141.0)	120.0 (100.0 - 140.5)	0.0019	0.0010	0.0441
Non-HDL cholesterol (mg/dL)	138.0 (116.0 - 161.0)	139.0 (117.0 - 163.0)	142.0 (119.0 - 162.0)	0.0045	0.0009	1.86×10^{-7}
HDL cholesterol (mg/dL)	53.0 (45.0 - 63.0)	53.0 (44.0 - 62.0)	52.0 (44.0 - 61.0)	-0.0059	0.0008	1.38×10^{-14}
Triglyceride (mg/dL)	90.0 (63.0 - 133.0)	94.0 (66.0 - 138.0)	96.0 (69.0 - 146.5)	0.0179	0.0018	2.12×10^{-23}
Remnant cholesterol (mg/dL)	16.0 (11.0 - 23.0)	17.0 (12.0 - 24.0)	17.0 (12.0 - 23.5)	0.0182	0.0022	1.33×10^{-16}
Metabolic syndrome (%)	25.34% (25,002)	26.59% (4064)	30.53% (167)	0.1174	0.0212	3.24×10^{-8}

Abbreviations and participant recruitment as in Table 2.
#Level was presented as median (interquartile range).
p value: adjusted for sex, age, BMI and current smoking.

Supplementary Table S3. Association between *APOB* rs693 genotypes and serum lipid levels and metabolic syndrome.

APOB rs693	GG (96,479)	GA (9777)	AA (231)	Beta	se	*p* value
Total cholesterol# (mg/dL)	193.0 (171.0 - 217.0)	194.0 (172.0 - 219.0)	199.0 (176.0 - 222.0)	0.0039	0.0008	5.53×10^{-7}
LDL cholesterol# (mg/dL)	119.0 (99.0 - 140.0)	121.0 (101.0 - 142.0)	125.0 (104.0 - 143.0)	0.0066	0.0012	1.21×10^{-8}
Non-HDL cholesterol# (mg/dL)	138.0 (116.0 - 161.0)	140.0 (118.0 - 164.0)	143.0 (119.0 - 168.0)	0.0082	0.0010	3.52×10^{-15}
HDL cholesterol# (mg/dL)	53.0 (45.0 - 63.0)	52.0 (45.0 - 62.0)	53.0 (43.0 - 64.0)	-0.0074	0.0009	1.47×10^{-15}
Triglyceride# (mg/dL)	90.0 (64.0 - 133.0)	94.0 (66.0 - 137.0)	92.0 (68.0 - 139.0)	0.0160	0.0022	1.16×10^{-13}
Remnant cholesterol# (mg/dL)	16.0 (11.0 - 23.0)	17.0 (12.0 - 24.0)	17.0 (12.0 - 24.0)	0.0170	0.0026	1.26×10^{-10}
Metabolic syndrome (%)	25.44% (26514)	26.49% (2813)	22.80% (57)	0.0798	0.0255	0.0018

Abbreviations, participant recruitment and adjustment as in Table 2.

Supplementary Table S4. Stepwise linear regression analysis by adding rs693 to other *APOB* variants in Table 3

	Total cholesterol				LDL-cholesterol				Non-HDL-cholesterol			
	Beta	se	R^2	*p* value	Beta	se	R^2	*p* value	Beta	se	R^2	*p* value
Age (years)	0.0013	0.0000	0.0340	$< 10^{-307}$	0.0014	0.0000	0.0168	$< 10^{-307}$	0.0019	0.0000	0.0352	$< 10^{-307}$
Sex (male vs. female)	0.0140	0.0005	0.0047	1.83E-171	-	-	-	-	-0.0052	0.0008	0.0007	4.22E-12
Body mass index (kg/m^2)	0.0016	0.0001	0.0053	1.23E-134	0.0050	0.0001	0.0258	$< 10^{-307}$	0.0061	0.0001	0.0485	$< 10^{-307}$
Current smoking (%)	-	-	-	-	-0.0028	0.0009	0.0204	1.51E-03	0.0031	0.0009	0.0001	6.31E-04
APOB rs693 (GG vs. GA vs. AA)	-	-	-	-	-	-	-	-	-	-	-	-
APOB rs144467873 (GG vs. GA vs. AA)	0.0754	0.0041	0.0034	9.42E-77	0.1290	0.0061	0.0303	5.56E-100	-0.0134	0.0006	0.0045	4.97E-97
APOB rs13306194 (GG vs. GA vs. AA)	-0.0093	0.0005	0.0041	2.38E-86	-0.0128	0.0007	0.0203	9.11E-74	0.1072	0.0055	0.0036	6.50E-85
APOB rs1367117 (GG vs. GA vs. AA)	0.0047	0.0005	0.0008	1.65E-21	0.0075	0.0007	0.0312	2.96E-24	0.0072	0.0007	0.0010	3.40E-27

	HDL-cholesterol				Triglyceride				Remnant cholesterol			
	Beta	se	R^2	*p* value	Beta	se	R^2	*p* value	Beta	se	R^2	*p* value
Age (years)	0.0001	0.0000	0.0002	8.90E-09	0.0030	0.0001	0.0194	$< 10^{-307}$	0.0050	0.0001	0.0419	$< 10^{-307}$
Sex (male vs. female)	0.0626	0.0007	0.0869	$< 10^{-307}$	-0.0568	0.0016	0.0200	1.20E-288	-0.0150	0.0019	0.0005	4.57E-15
Body mass index (kg/m^2)	-0.0094	0.0001	0.1645	$< 10^{-307}$	0.0221	0.0002	0.1504	$< 10^{-307}$	0.0099	0.0002	0.0230	$< 10^{-307}$
Current smoking (%)	-0.0109	0.0008	0.0013	4.91E-43	0.0408	0.0019	0.0036	9.95E-106	0.0316	0.0023	0.0033	1.00E-43
APOB rs693 (GG vs. GA vs. AA)	-0.0030	0.0011	0.0000	5.67E-03	-	-	-	-	-	-	-	-
APOB rs1318006 (AA vs. AG vs. GG)	-0.0053	0.0007	0.0008	1.93E-13								
APOB rs35131127 (TT vs. TC vs. CC)					0.0169	0.0016	0.0010	1.24E-24				
APOB rs13306194 (GG vs. GA vs. AA)					-0.0126	0.0013	0.0007	1.03E-21	-0.0147	0.0016	0.0009	5.33E-20
APOB rs144467873 (GG vs. GA vs. AA)									-0.0974	0.0140	0.0004	4.08E-12
APOB rs56213756 (CC vs. CG vs. GG)									0.0147	0.0017	0.0006	2.76E-17

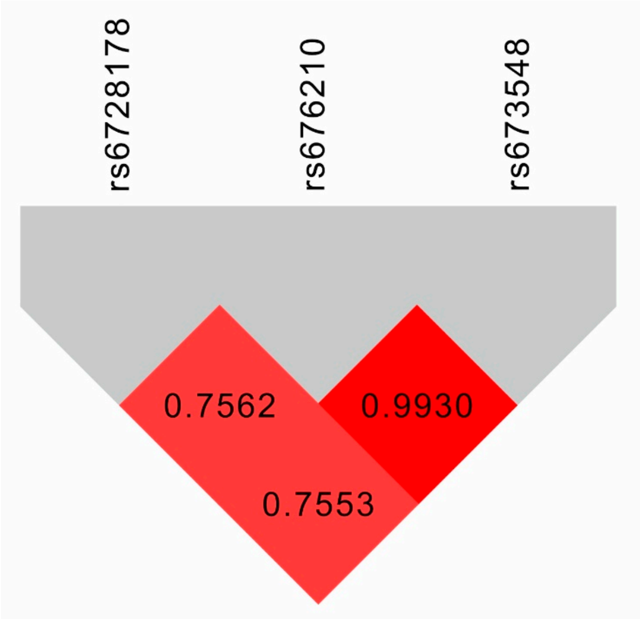

Supplementary Figure S2. Linkage disequilibrium maps for the *APOB* gene rs6728178, rs676210 and rs673548 variants.

Supplementary Table S5. Association between three *APOB* variants rs676210, rs673548, and rs6728178 genotypes and LDL cholesterol levels and the risk of metabolic syndrome in Taiwan Biobank participants.

Chr	SNPs	Position(GRCh37)	Ref/Alt	LDL cholesterol (mg/dL)			metabolic syndrome		
				Beta	se	*p* value	OR	95% CI	*p* value
2	rs676210	21231524	G/A	-0.0063	0.0006	4.10×10^{-30}	1.0214	0.9968 - 1.0466	0.0891
2	rs673548	21237544	G/A	-0.0063	0.0006	7.47×10^{-30}	1.0221	0.9975 - 1.0473	0.0785
2	rs6728178	21193946	G/A	-0.0052	0.0006	5.32×10^{-21}	1.0214	0.9971 - 1.0463	0.0850

Participant recruitment for analysis is shown in Fig. 1

p value: adjusted for sex, age, BMI and current smoking.

Abbreviation as in Table 4 and Supplementary Table 1.

Supplementary Table S6. Association between *APOB* rs4665709 genotypes and clinical and laboratory parameters in Taiwan Biobank participants.

Clinical and laboratory parameters	Beta	se	*p* value
Anthropology			
Age (years)	0.0264	0.0887	0.7656
Waist circumference (cm)	0.0013	0.0412	0.9754
Waist-hip ratio	0.0001	0.0004	0.9058
Body mass index (kg/m^2)	-0.0372	0.0301	0.2168
Blood Pressure status			
Systolic BP* (mmHg)	0.0823	0.1242	0.5076
Diastolic BP* (mmHg)	0.1278	0.0812	0.1156
Mean BP* (mmHg)	0.1126	0.0893	0.2071
Lipid profile			
Total cholesterol# (mg/dL)	0.0016	0.0006	0.0133
LDL cholesterol# (mg/dL)	0.0019	0.0010	0.0441
Non-HDL cholesterol# (mg/dL)	0.0045	0.0009	1.86×10^{-7}
HDL cholesterol# (mg/dL)	-0.0059	0.0008	1.38×10^{-14}
Triglyceride# (mg/dL)	0.0179	0.0018	2.12×10^{-23}
Remnant cholesterol# (mg/dL)	0.0182	0.0022	1.33×10^{-16}
Glucose metabolism			
Fasting plasma glucose** (mg/dL)	0.0560	0.1212	0.6438
HbA1c** (%)	0.0039	0.0048	0.4188
Uric acid			
Uric acid*** (mg/dL)	-0.0107	0.0090	0.2369
Renal function			
Creatinine (mg/dL)	-0.0062	0.0023	0.0061
eGFR (mL/min/1.73 m^2)	0.3880	0.1755	0.0271
BUN (mg/dL)	-0.0387	0.0294	0.1883
Liver function			
AST (U/L)	0.0349	0.0989	0.7243
ALT (U/L)	0.0417	0.1611	0.7958
γGT (U/L)	0.2710	0.2583	0.2940
Serum albumin (g/dL)	-0.0012	0.0018	0.4915
Total bilirubin (mg/dL)	0.0006	0.0022	0.7839
Hematological parameters			
Leukocyte count (10^3/μL)	0.5855	0.2419	0.0155
Hematocrit (%)	-0.0239	0.0279	0.3928
Platelet count (10^3/μL)	-0.1803	0.4734	0.7033
Red blood cell count (10^6/μL)	-0.0010	0.0037	0.7745
Hemoglobin (g/dL)	0.0060	0.0101	0.5543
Atherosclerotic risk factors			
Diabetes mellitus (%)	0.0677	0.0288	0.0187
Hypertension (%)	-0.0022	0.0218	0.9194
Current smoking (%)	-0.0366	0.0232	0.1148
Gout (%)	-0.0004	0.0437	0.9919
Microalbuminuria (%)	0.0002	0.0262	0.9949
Metabolic syndrome (%)	0.1174	0.0212	3.24×10^{-8}

Abbreviations: AST: aspartate aminotransferase, ALT: alanine aminotransferase, γGT: γ-glutamyl transferase, eGFR: estimated glomerular filtration rate, BUN: blood urine nitrogen, HbA1c: hemoglobin A1C, BP: blood pressure.
Other abbreviations as in Table 1. Participants were analyzed after the exclusion of those with a history of *hypertension, **diabetes mellitus, ***gout, and #hyperlipidemia. *p* value: adjusted for sex, age, BMI and current smoking.

Supplementary Table S7. Association between *APOB* rs144467873 genotypes and clinical and laboratory parameters in Taiwan Biobank participants.

Clinical and laboratory parameters	Beta	se	*p* value
Anthropology			
Age (years)	-0.1601	0.5193	0.7579
Waist circumference (cm)	0.0376	0.2411	0.8760
Waist-hip ratio	0.0017	0.0025	0.5015
Body mass index (kg/m^2)	-0.2928	0.1762	0.0966
Blood Pressure status			
Systolic BP* (mmHg)	-0.0999	0.7201	0.8897
Diastolic BP* (mmHg)	-0.8174	0.4710	0.0826
Mean BP* (mmHg)	-0.5785	0.5177	0.2638
Lipid profile			
Total cholesterol# (mg/dL)	0.0805	0.0041	2.29×10^{-85}
LDL cholesterol# (mg/dL)	0.1373	0.0061	1.96×10^{-110}
Non-HDL cholesterol# (mg/dL)	0.1148	0.0055	6.08×10^{-95}
HDL cholesterol# (mg/dL)	-0.0124	0.0049	0.0114
Triglyceride# (mg/dL)	-0.0077	0.0115	0.5037
Remnant cholesterol# (mg/dL)	-0.1005	0.0142	1.71×10^{-12}
Glucose metabolism			
Fasting plasma glucose** (mg/dL)	-0.6737	0.7071	0.3407
HbA1c** (%)	-0.0015	0.0282	0.9562
Uric acid			
Uric acid*** (mg/dL)	0.0080	0.0529	0.8793
Renal function			
Creatinine (mg/dL)	-0.0108	0.0132	0.4135
eGFR (mL/min/1.73 m^2)	1.0942	1.0283	0.2873
BUN (mg/dL)	-0.3357	0.1725	0.0517
Liver function			
AST (U/L)	-0.1482	0.5780	0.7977
ALT (U/L)	0.0103	0.9424	0.9913
γGT (U/L)	-1.9279	1.5212	0.2050
Serum albumin (g/dL)	-0.0183	0.0106	0.0856
Total bilirubin (mg/dL)	-0.0223	0.0130	0.0855
Hematological parameters			
Leukocyte count (10^3/μL)	-0.1318	1.4123	0.9256
Hematocrit (%)	-0.2059	0.1635	0.2079
Platelet count (10^3/μL)	-0.3013	2.7717	0.9134
Red blood cell count (10^6/μL)	-0.0088	0.0214	0.6812
Hemoglobin (g/dL)	-0.0686	0.0592	0.2468
Atherosclerotic risk factors			
Diabetes mellitus (%)	0.0775	0.1711	0.6507
Hypertension (%)	-0.0181	0.1289	0.8884
Current smoking (%)	-0.1831	0.1376	0.1835
Gout (%)	0.0374	0.2519	0.8821
Microalbuminuria (%)	-0.0811	0.1611	0.6148
Metabolic syndrome (%)	0.6463	0.1161	2.58×10^{-8}

Abbreviations, participant recruitment and adjustment as in Supplementary Table 6.

Supplementary Table S8. F-statistics for *APOB* variants.

Chr	Gene	SNP	position	Allele	MAF	HWE	beta	SE	*P* value*	F-statistic	R^2
2	*APOB*	rs144467873	21229161	G/A	0.0019	0.2916	0.1373	0.0061	1.96×10^{-110}	1071.5	0.0470
2	*APOB*	rs13306194	21252534	G/A	0.1425	0.04943	-0.0140	0.0007	5.13×10^{-89}	1039.5	0.0463
2	*APOB*	rs1367117	21263900	G/A	0.1320	0.8006	0.0109	0.0007	5.52×10^{-50}	1015.0	0.0446
	WGRS_*APOB*_3SNPs						0.8796	0.0277	5.81×10^{-221}	1160.3	0.0517
	WGRS_*APOB*_2SNPs						0.8805	0.0375	9.08×10^{-122}	1070.2	0.0476

*LDL-C adjusted for sex, age, body mass index and current smoking
